# Generalizability of Treatment Outcome Prediction Across Antidepressant Treatment Trials in Depression

**DOI:** 10.1001/jamanetworkopen.2025.1310

**Published:** 2025-03-20

**Authors:** Peter Zhukovsky, Madhukar H. Trivedi, Myrna Weissman, Ramin Parsey, Sidney Kennedy, Diego A. Pizzagalli

**Affiliations:** 1 Center for Depression, Anxiety and Stress Research, Department of Psychiatry, McLean Hospital, Harvard Medical School, Belmont, Massachusetts; 2 Department of Psychiatry, University of Texas, Southwestern Medical Center, Dallas; 3 Department of Psychiatry, New York State Psychiatric Institute, New York, New York; 4 Columbia University Vagelos College of Physicians and Surgeons, New York, New York; 5 Department of Psychiatry, Stony Brook University, Stony Brook, New York; 6 Department of Psychiatry, University of Toronto, Toronto, Ontario, Canada; 7 Centre for Depression and Suicide Studies, Unity Health Toronto, Toronto, Ontario, Canada

## Abstract

**Importance:**

Although several predictive models for response to antidepressant treatment have emerged on the basis of individual clinical trials, it is unclear whether such models generalize to different clinical and geographical contexts.

**Objective:**

To assess whether neuroimaging and clinical features predict response to sertraline and escitalopram in patients with major depressive disorder (MDD) across 2 multisite studies using machine learning and to predict change in depression severity in 2 independent studies.

**Design, Setting, and Participants:**

This prognostic study included structural and functional resting-state magnetic resonance imaging and clinical and demographic data from the Establishing Moderators and Biosignatures of Antidepressant Response in Clinical Care (EMBARC) randomized clinical trial (RCT), which administered sertraline (in stage 1 and stage 2) and placebo, and the Canadian Biomarker Integration Network in Depression (CANBIND-1) RCT, which administered escitalopram. EMBARC recruited participants with MDD (aged 18-65 years) at 4 academic sites across the US between August 2011 and December 2015. CANBIND-1 recruited participants with MDD from 6 outpatient centers across Canada between August 2013 and December 2016. Data were analyzed from October 2023 to May 2024.

**Main Outcomes and Measures:**

Prediction performance for treatment response was assessed using balanced classification accuracy and area under the curve (AUC). In secondary analyses, prediction performance was assessed using observed vs predicted correlations between change in depression severity.

**Results:**

In 363 adult patients (225 from EMBARC and 138 from CANBIND-1; mean [SD] age, 36.6 [13.1] years; 235 women [64.7%]), the best-performing models using pretreatment clinical features and functional connectivity of the dorsal anterior cingulate had moderate cross-trial generalizability for antidepressant treatment (trained on CANBIND-1 and tested on EMBARC, AUC = 0.62 for stage 1 and AUC = 0.67 for stage 2; trained on EMBARC stage 1 and tested on CANBIND-1, AUC = 0.66). The addition of neuroimaging features improved the prediction performance of antidepressant response compared with clinical features only. The use of early-treatment (week 2) instead of pretreatment depression severity scores resulted in the best generalization performance, comparable to within-trial performance. Multivariate regressions showed substantial cross-trial generalizability in change in depression severity (predicted vs observed *r* ranging from 0.31 to 0.39).

**Conclusions and Relevance:**

In this prognostic study of depression outcomes, models predicting response to antidepressants show substantial generalizability across different RCTs of adult MDD.

## Key Points

**Question:**

Can neuroimaging and clinical features predict response to sertraline and escitalopram in patients with major depressive disorder across 2 large multisite studies?

**Findings:**

In this prognostic study of depression outcomes, among 363 patients in 2 trials, the best-performing models using pretreatment clinical features and functional connectivity of the dorsal anterior cingulate showed substantial cross-trial generalizability. The addition of neuroimaging features significantly improved prediction performance of antidepressant response compared with models including only clinical features.

**Meaning:**

Promising generalizability of depression response markers emerged across 2 independent clinical trials of adults with major depressive disorder; future studies with improved predictive models are needed to optimize treatment outcomes.

## Teaser

This prognostic study examines whether neuroimaging and clinical features predict response to sertraline and escitalopram in patients with major depressive disorder across 2 multisite studies using machine learning.

## Introduction

Treatment of psychiatric conditions, including major depressive disorder (MDD) often fails, with more than one-half of patients with MDD not responding to first-line antidepressant treatment.^1^ Leveraging machine learning in prediction of treatment response promises to accelerate symptom reduction. However, a recent study^2^ of clinical markers predicting treatment outcomes has highlighted the challenge of identifying markers that generalize across trials. Although several studies^3,4,5,6,7^ have identified promising markers of antidepressant response, it is unclear whether findings generalize across trials and, thus, to future patients.

Clinical trials featuring biomarkers alongside in-depth clinical assessments are scarce and expensive, and different trials often use distinct assessments and imaging protocols to evaluate potential biomarkers.^2,8^ As a result, there are no markers of treatment response that cut across different MDD treatment trials,^8^ although higher pretreatment depression severity and early change in depression scores have been linked to treatment response.^4^

Functional connectivity (FC) is an important neuroimaging predictor of antidepressant response in MDD.^8,9,10,11^ Systematic reviews have identified a number of regions whose FC may be associated with response to pharmacological and neurostimulation treatments, including dorsolateral and ventrolateral prefrontal cortex, lateral parietal areas, and the anterior cingulate cortex (ACC).^8,9^ Response to selective serotonin reuptake inhibitors (SSRIs) in particular has been associated with lower connectivity between the ACC with the dorsolateral prefrontal cortex (dlPFC) and insula.^6^ Among structural markers of response, reduced gray matter volume in cortical regions^12^ and hippocampus^13^ predicted treatment outcomes, especially in late-life depression. Critically, recent studies^14,15^ linking magnetic resonance imaging (MRI) data with maps of gene expression and receptor binding can help improve interpretation of molecular correlates of MRI-derived biomarkers.

Clinical and pathophysiological heterogeneity may explain variability in MDD biomarkers.^9^ To address heterogeneity, unique biomarkers need to be identified in independent samples. Accordingly, studies combining clinical trials of different samples, similar to Chekroud et al,^2^ are needed to test the generalizability and robustness of clinical and biological markers of treatment outcomes. Accordingly, we investigated the generalizability of models featuring clinical and functional MRI (fMRI) features across 2 MDD trials, the Establishing Moderators and Biosignatures of Antidepressant Response in Clinical Care (EMBARC) and Canadian Biomarker Integration Network in Depression (CANBIND-1). These trials administered SSRIs—sertraline in EMBARC and escitalopram in CANBIND-1—to adults with non–treatment-resistant MDD. We expected early-treatment models to outperform pretreatment models and the addition of ACC connectivity features to improve model performance.

## Methods

### Study Design

In this prognostic study, we used clinical, demographic, and neuroimaging data from 2 MDD randomized clinical trials: EMBARC and CANBIND-1. EMBARC is a 2-stage trial that recruited participants with MDD (aged 18-65 years) at 4 academic sites across the US between August 2011 and December 2015; participants were randomized to sertraline or placebo in stage 1. After 8 weeks, in stage 2, nonresponders to sertraline were switched to bupropion, nonresponders to placebo were switched to sertraline, and responders to sertraline or placebo continued with their treatment. Similarly, CANBIND-1 is a 2-step trial that recruited participants with MDD from 6 outpatient centers across Canada between August 2013 and December 2016; participants received escitalopram in stage 1 for 8 weeks. In stage 2, nonresponders were offered an augmentation treatment of escitalopram with aripiprazole, whereas responders continued with their treatment. Detailed descriptions of the EMBARC^16^ and CANBIND-1^17,18^ design have been published elsewhere. Ethical approval for EMBARC was obtained from the institutional review board at each site. Approval for CANBIND-1 was obtained from research ethics boards at each site. Participants provided written, informed consent for all study procedures. The methods of the current study follow Transparent Reporting of a Multivariable Prediction Model for Individual Prognosis or Diagnosis (TRIPOD) reporting guidelines.

### Participants

Among 296 unmedicated outpatients in EMBARC, we defined 3 subgroups: individuals treated with sertraline in stage 1 with complete data, a different group of individuals treated with sertraline in stage 2 after not responding to placebo, and individuals receiving placebo in stage 1. The stage 2 sertraline group is, thus, a subset of the stage 1 placebo group, who did not respond to placebo. Among 144 patients with MDD in CANBIND-1, we included those who had completed at least 6 weeks of escitalopram treatment and had complete baseline prediction data.

### Clinical Data

In addition to age, sex, employment status, and body mass index (BMI; calculated as weight in kilograms divided by height in meters squared), we used baseline depression severity (CANBIND-1, Montgomery Asberg Depression Rating Scale [MADRS]^19^ and EMBARC, 17-item Hamilton Depression Rating Scale [HDRS]^20^), and anhedonia (Snaith Hamilton Rating Scale [SHAPS]^21^) as baseline predictors. MADRS scores were converted to HDRS in CANBIND-1 following a validated mapping^22^ (eAppendix 1 in Supplement 1). Early treatment models included the change in depression scores between week 2 and baseline.

### Treatment Outcomes

Primary treatment outcome was treatment response, defined as a 50% or greater reduction in depression severity (EMBARC, HDRS scores; CANBIND-1, converted MADRS scores). In EMBARC, HDRS was assessed at weeks 8 and 16 in stages 1 and 2, respectively, whereas MADRS outcomes were collected at week 8 in CANBIND-1. When no data were available at week 8 in CANBIND-1, the closest assessment (ie, week 6) data were used instead. We also present analyses predicting change in depression severity in eAppendix 2 in Supplement 1.

### MRI Data

We preprocessed structural and resting-state fMRI data in EMBARC using fMRIPrep software version 22.1.1 and in CANBIND-1 using fMRIPrep software version 23.0.2 (The fMRIPrep Developers), using fixed confound regression for denoising and applied a 6-mm smoothing kernel. FC matrices were calculated for the Human Connectome Project cortical parcellation.^23^ Global cortical FC was obtained by averaging the rows of these connectivity matrices; we also obtained seed-based FC of the dorsal ACC (dACC) and rostral ACC (rACC), respectively.

### Statistical Analysis

Data were analyzed from October 2023 to May 2024. Following recent studies,^2^ we used elastic net logistic regressions with regularization (lassoglm in Matlab R2022a^24^; MathWorks) to predict treatment outcomes in the 4 datasets. We tested 5 sets of models using baseline depression severity: (1) a clinical model including age, sex, employment, baseline HRSD, SHAPS, and BMI; (2) a clinical plus global FC model; (3) a clinical plus dACC FC model; (4) a clinical plus rACC FC model; and (5) a clinical plus cortical thickness model. We then tested 5 analogous models that included week 2 instead of baseline depression severity scores. We provide more details on predictive modeling in eAppendix 2 in Supplement 1. The baseline models included the following: (1) response predicted by age plus sex plus employment plus baseline HDRS plus SHAPS plus BMI; (2) response predicted by age plus sex plus employment plus baseline HDRS plus SHAPS plus BMI plus global FC; (3) response predicted by age plus sex plus employment plus baseline HDRS plus SHAPS plus BMI plus dACC FC; (4) response predicted by age plus sex plus employment plus baseline HDRS plus SHAPS plus BMI plus rACC FC; and (5) response predicted by age plus sex plus employment plus baseline HDRS plus SHAPS plus BMI plus brain structure

First, we tested the prediction performance of models within CANBIND-1 and EMBARC stage 1 by creating 100 random training and test data splits, training the elastic net model with 10-fold cross-validation on the training dataset, and predicting outcomes in the test dataset on each iteration. Second, we evaluated the prediction performance of models trained on CANBIND-1 and tested in EMBARC stage 1, EMBARC stage 2, and EMBARC placebo. Area under the curve (AUC) and balanced accuracy^2^ were used to assess prediction performance. Across all models, we used thresholds derived from simulation studies to assess whether balanced accuracy was significantly higher than chance (*P* < .05).^3,25^ Second, for a select subset of models (clinical features only and clinical plus dACC models), bootstrapping was used to test whether AUCs were significantly higher than chance and to compare the most promising models with each other (1-tailed *P* < .05).

Next, in secondary analyses we used a multivariate partial least squares regression (PLS-R) to predict change in depression severity scores after treatment (eAppendix 2 in Supplement 1). PLS-R predictors included 360 dACC connectivity features, age, sex, employment, baseline HDRS-17, SHAPS, and BMI. We used permutation testing (n = 5000) to assess model significance (permutation *P* < .05) and bootstrapping (n = 5000; |Z| > 3) to identify robust features. After training the model on CANBIND-1, we applied the regression weights to predict change in depression severity in EMBARC. Similarly, after training the model on EMBARC stage 1 sertraline, we applied the regression weights to CANBIND-1, EMBARC stage 2 sertraline, and EMBARC stage 1 placebo.

In sensitivity analyses, first, we reanalyzed the data while correcting for batch effects in the resting-state fMRI data and the gray matter brain structure using ComBat.^26^ Second, we bootstrapped elastic net models with the full set of 366 predictors. All code is available elsewhere.^27^

## Results

An overview of the demographic and clinical features of the participant groups is provided in Table 1. Briefly, of the 363 participants (225 from EMBARC and 138 from CANBIND-1; mean [SD] age, 36.6 [13.1] years), 235 (64.7%) were women, and they showed response rates ranging between 40% and 60%. A detailed breakdown of participant drop-out and missing data is provided in eFigure 1 in Supplement 1. We evaluated the out-of-trial performance of 10 machine learning models in 2 training scenarios: first, we trained the models in CANBIND-1 and tested them in EMBARC stage 1 sertraline, stage 2 sertraline, and stage 1 placebo conditions; second, we trained the models in EMBARC stage 1 sertraline and tested them in CANBIND-1 and EMBARC stage 2 sertraline, as well as EMBARC stage 1 placebo conditions.

**Table 1.** Demographic and Clinical Sample Characteristics

Characteristic	Participants, No. (%)			
	CANBIND-1 escitalopram (n = 138)	EMBARC stage 1 sertraline (n = 110)	EMBARC stage 2 sertraline (n = 60)^a^	EMBARC stage 1 placebo (n = 115)
Sex				
Female	89 (64.5)	75 (68.2)	34 (56.7)	71 (61.7)
Male	49 (35.5)	35 (31.8)	26 (43.3)	44 (38.3)
Employed	65 (47.1)	62 (56.4)	30 (50.0)	63 (54.8)
Race				
African American or Black	5 (3.6)	22 (20.0)	10 (16.7)	18 (15.7)
Asian	19 (13.8)	5 (4.5)	2 (3.3)	8 (6.9)
White	106 (76.8)	73 (66.4)	43 (71.7)	80 (69.7)
Other^b^	12 (8.7)	10 (9.1)	5 (8.3)	9 (7.8)
Ethnicity				
Hispanic	9 (6.5)	20 (18.2)	10 (16.7)	20 (17.4)
Non-Hispanic	129 (93.5)	90 (81.8)	50 (83.3)	95 (82.6)
Response	60 (43.5)	58 (52.7)	36 (60.0)	42 (36.5)
Age, mean (SD), y	34.8 (12.4)	38.2 (14.0)	39.2 (13.3)	37.3 (13.1)
Body mass index, mean (SD)^c^	26.3 (5.9)	28.7 (8.1)	27.5 (5.9)	28.2 (6.8)
Years of education, mean (SD)	16.9 (2.1)	15.1 (2.6)	15.3 (2.5)	15.4 (2.4)
Pretreatment depression severity, mean (SD)^d^	22.1 (4.3)	18.7 (4.4)	18.6 (3.8)	18.7 (4.3)
Snaith Hamilton Rating Scale, mean (SD)	35.6 (6.0)	33.5 (5.4)	33.2 (5.4)	33.2 (5.8)

Abbreviations: CANBIND-1, Canadian Biomarker Integration Network in Depression; EBARC, Establishing Moderators and Biosignatures of Antidepressant Response in Clinical Care.

^a^ Participants who received sertraline in EMBARC stage 2 had not responded to placebo in stage 1.

^b^ Other race refers to multiracial or unknown. In CANBIND-1 participants could indicate multiple races at the same time.

^c^ Body mass index is calculated as weight in kilograms divided by height in meters squared.

^d^ Pretreatment depression severity was assessed using the 17-item Hamilton Depression Rating Scale in EMBARC and scores converted from the Montgomery Asberg Depression Rating Scale to the Hamilton Depression Rating Scale scores in CANBIND-1.

### Pretreatment Models of Response

**Figure 1.**
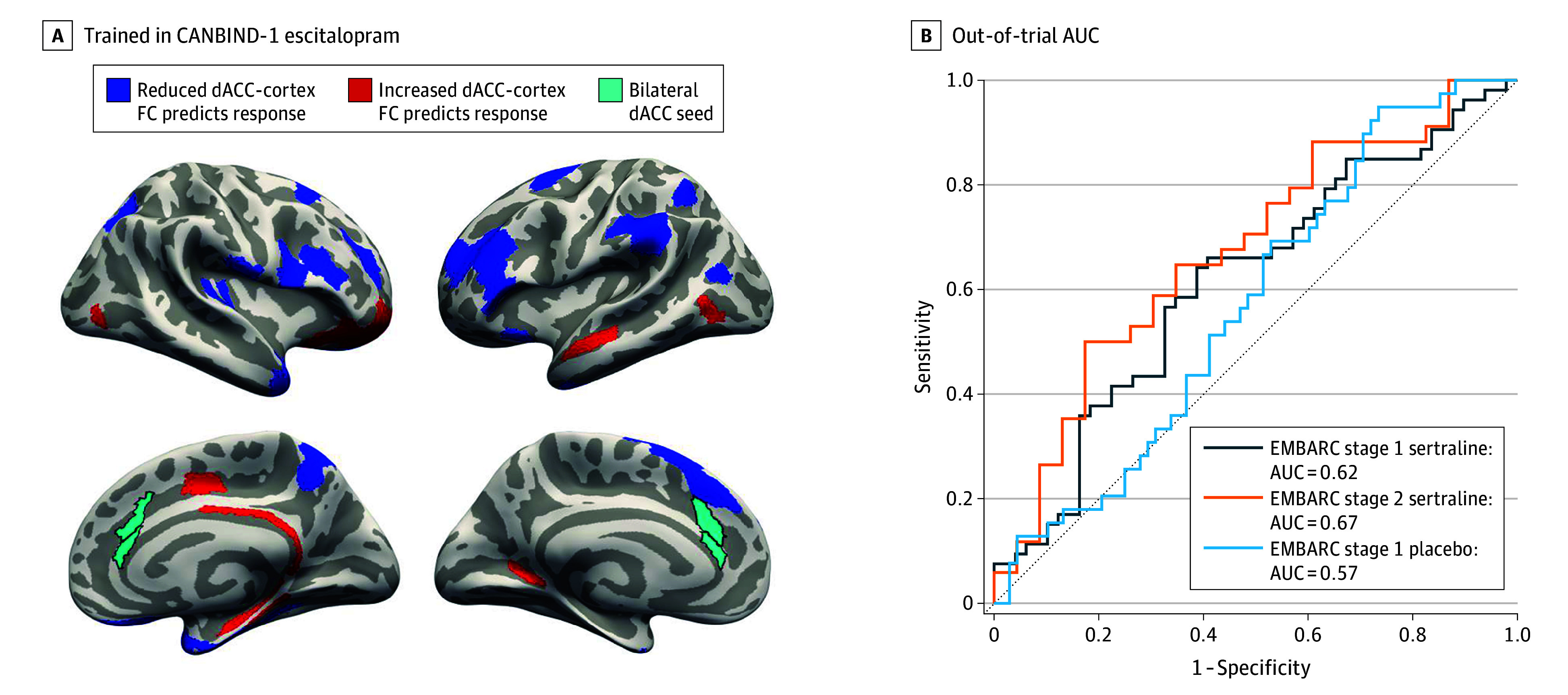
Functional Connectivity (FC) Predictors of Treatment Response in Canadian Biomarker Integration Network in Depression (CANBIND-1) and the Out-of-Trial Generalization Performance We trained models on CANBIND-1 escitalopram data and then tested them on Establishing Moderators and Biosignatures of Antidepressant Response in Clinical Care (EMBARC) stage 1 sertraline, EMBARC stage 2 sertraline, and EMBARC stage 1 placebo groups. We show the seed-based dorsal anterior cingulate (dACC) connectivity maps predicting response in CANBIND-1 (A) alongside the respective out-of-trial area under the receiver operator curve (AUC) analyses (B). The dACC seed highlighted in light green (A) was selected on the basis of the overlap between the global FC maps in CANBIND-1 (eFigure 2 in Supplement 1) and prior literature on the ACC involvement in major depression.

**Figure 2.**
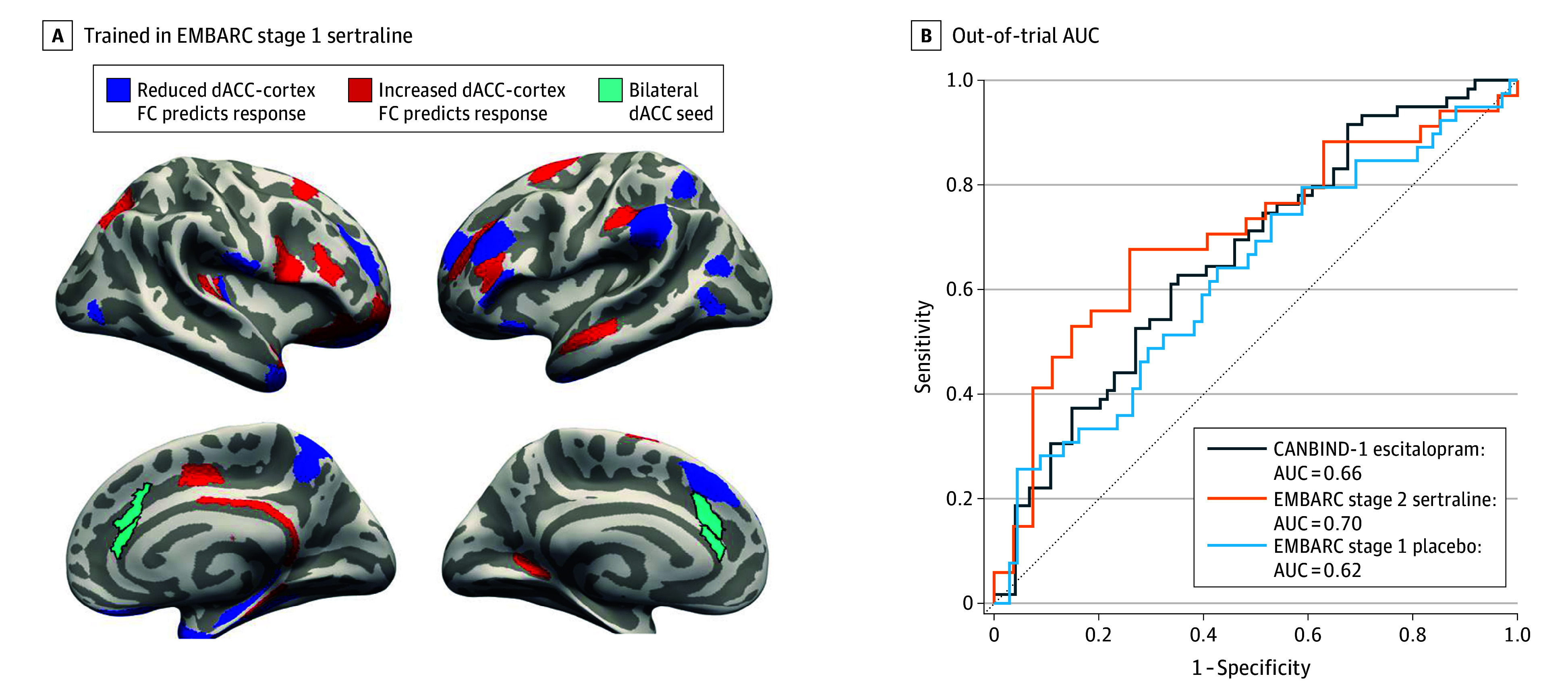
Functional Connectivity (FC) Predictors of Treatment Response in Establishing Moderators and Biosignatures of Antidepressant Response in Clinical Care (EMBARC) and the Out-of-Trial Generalization Performance We trained models on the EMBARC stage 1 sertraline sample and tested the resulting models on Canadian Biomarker Integration Network in Depression (CANBIND-1) escitalopram, EMBARC stage 2 sertraline, and EMBARC stage 1 placebo samples. We show the seed-based dorsal anterior cingulate (dACC) connectivity maps (A) predicting response in EMBARC alongside the respective out-of-trial area under the receiver-operator curve (AUC) analyses (B). The dACC seed highlighted in light green (A) was selected on the basis of the overlap between the global FC maps in CANBIND-1 (eFigure 2 in Supplement 1) and prior literature on the ACC involvement in major depression.

AUC and balanced accuracy values for pretreatment models are summarized in Table 2. We found that the clinical data model and the model using dACC-to-cortex connectivity alongside clinical data performed best (trained on CANBIND-1 and tested on EMBARC, AUC = 0.62 for stage 1 and AUC = 0.67 for stage 2; trained on EMBARC stage 1 and tested on CANBIND-1, AUC = 0.66). Although the clinical model reached out-of-trial AUCs of 0.58 to 0.61 and balanced accuracy of 59% to 61% when trained and tested on CANBIND-1 and EMBARC antidepressant groups, the addition of dACC connectivity features (clinical plus dACC) improved pairwise out-of-trial model performance to AUCs of 0.61 to 0.68 and balanced accuracy of 61% to 71%. Although the clinical and rACC connectivity model (clinical plus rACC) and the clinical and brain structure (clinical plus cortical thickness) model also achieved good performance when trained in CANBIND-1, they did not generalize well when trained in EMBARC stage 1. The addition of global FC features (clinical plus global FC) did not improve model performance, with worse AUC values across all training and testing setups for groups given SSRIs. Bootstrapping the models showed that the addition of dACC connectivity data significantly improved model performance for EMBARC stage 2 when the models were trained on CANBIND-1, with a trend in improvement in model performance for EMBARC stage 1 sertraline. When mapping the global FC predictors of response in CANBIND-1 and EMBARC (eFigure 2 in Supplement 1), we found a different pattern of connectivity that did not generalize across trials or within EMBARC. However, we found that lower connectivity of the dACC with dlPFC, medial temporal and parietal areas and higher dACC connectivity with the posterior cingulate (Figure 1A and Figure 2A) were predictive of response to antidepressants, generalizing across trials (Figure 1B and Figure 2B).

**Table 2.** Summary of Out-of-Trial Model Performance for Models Trained in the CANBIND-1 and EMBARC Clinical Trials^a^

Models of response	Models trained on CANBIND-1						Models trained on EMBARC stage 1 sertraline					
	Tested on EMBARC stage 1 sertraline		Tested on EMBARC stage 2 sertraline		Tested on EMBARC stage 1 placebo		Tested on CANBIND-1 escitalopram		Tested on EMBARC stage 2 sertraline		Tested on EMBARC stage 1 placebo	
	AUC	BA	AUC	BA	AUC	BA	AUC	BA	AUC	BA	AUC	BA
Pretreatment												
Clinical model^b^	0.58	0.61^c^	0.58	0.60^c^	0.63	0.59^c^	0.59	0.59^c^	0.61	0.60^c^	0.52	0.55
Clinical plus global FC^d^	0.56	0.59^c^	0.50	0.51	0.62	0.59^c^	0.52	0.56	0.60	0.59	0.49	0.57
Clinical plus dACC FC^d^	0.62	0.63^c^	0.67	0.65^c^	0.57	0.55	0.66	0.64^c^	0.70	0.71^c^	0.62	0.61^c^
Clinical plus rACC FC^d^	0.59	0.60^c^	0.63	0.65^c^	0.68	0.64^c^	0.51	0.52	0.57	0.56	0.44	0.46
Clinical plus CT^e^	0.58	0.56	0.61	0.63^c^	0.63	0.63^c^	0.56	0.52	0.62	0.62^c^	0.48	0.51
Early treatment												
Clinical model^f^	0.68	0.69^c^	0.73	0.66^c^	0.71	0.66^c^	0.66	0.69^c^	0.68	0.66^c^	0.63	0.66^c^
Clinical plus global FC^d^	0.64	0.64^c^	0.65	0.60^c^	0.69	0.64^c^	NV^g^	NV^g^	NV^g^	NV^g^	NV^g^	NV^g^
Clinical plus dACC FC^d^	0.68	0.64^c^	0.79	0.73^c^	0.70	0.69^c^	0.59	0.58^c^	0.71	0.70^c^	0.64	0.63^c^
Clinical plus rACC FC^d^	0.66	0.67^c^	0.74	0.67^c^	0.73	0.69^c^	NV^g^	NV^g^	NV^g^	NV^g^	NV^g^	NV^g^
Clinical plus CT^e^	0.69	0.69^c^	0.73	0.70^c^	0.73	0.69^c^	0.57	0.57	0.66	0.61^c^	0.56	0.51

Abbreviations: AUC, area under the curve; BA, balanced accuracy; dACC, dorsal anterior cingulate cortex; FC, functional connectivity; CT, cortical thickness; NV, no variables; rACC, rostral anterior cingulate cortex.

^a^ We analyzed data from 138 participants in CANBIND-1, 110 participants who received sertraline in stage 1 of EMBARC, 115 participants who received placebo in stage 1 of EMBARC, and 60 participants who received sertraline in EMBARC stage 2 after not responding to placebo in EMBARC stage 1.

^b^ Model includes age, sex, Snaith Hamilton Rating Scale score, employment, body mass index, and baseline Hamilton Depression Rating Scale and Montgomery Asberg Depression Rating Scale scores.

^c^ Balanced accuracy values were significantly higher than chance (*P* < .05, not correcting for the number of models) based on prior simulations.^25^

^d^ FC models trained on EMBARC stage 1 used variables derived from the CANBIND-1 models.

^e^ CT models trained on EMBARC stage 1 used variables derived from the CANBIND-1 models.

^f^ Model includes age, sex, Snaith Hamilton Rating Scale score, employment, body mass index, baseline Hamilton Depression Rating Scale and Montgomery Asberg Depression Rating Scale scores, and change in Hamilton Depression Rating Scale and Montgomery Asberg Depression Rating Scale scores at week 2.

^g^ No variables survive regularization.

### Early-Treatment Models of Response

**Figure 3.**
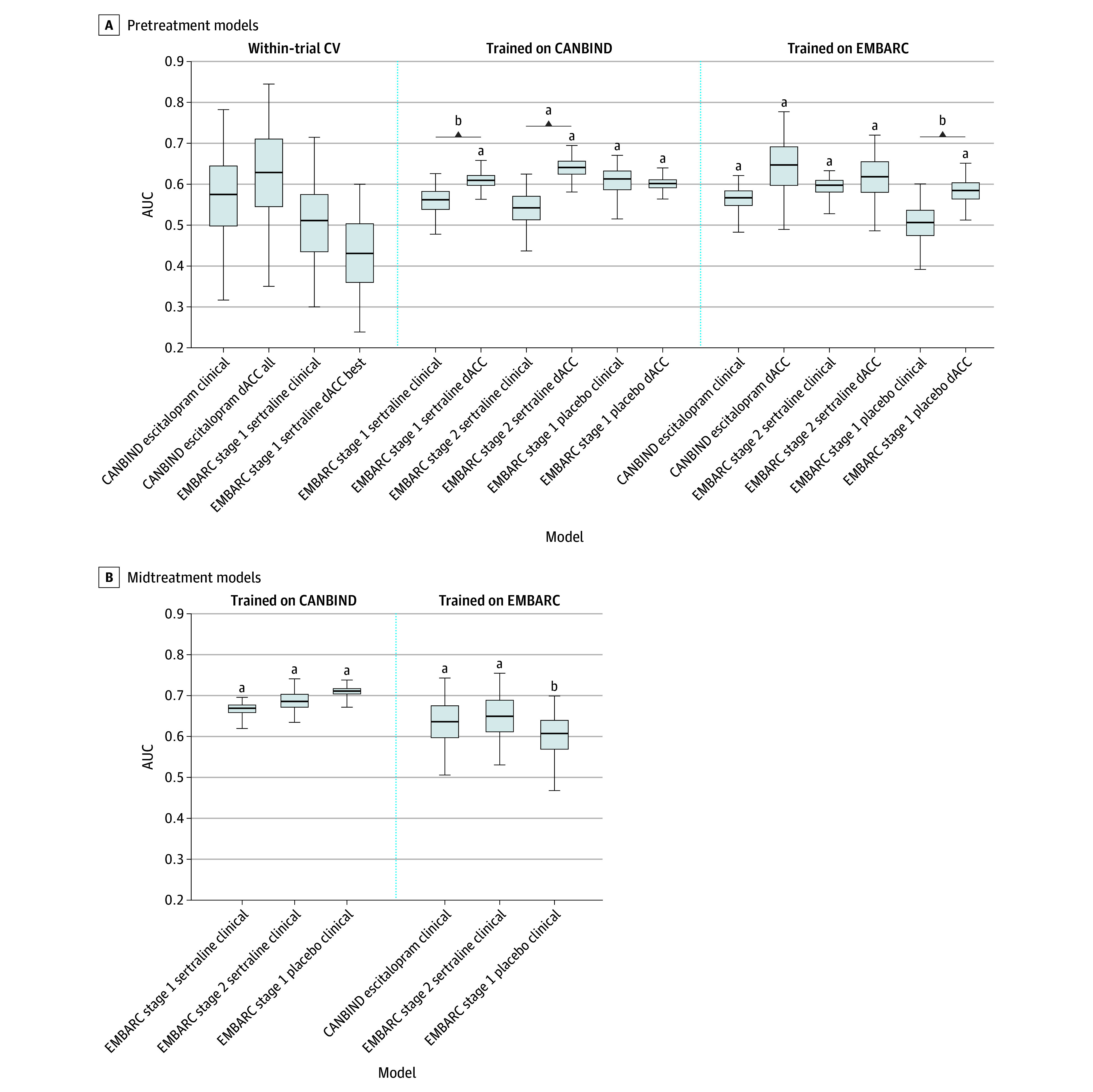
Area Under the Curve (AUC) for Models Predicting Treatment Response Across Best-Performing Models Derived From Bootstrapping Analyses Models trained on Canadian Biomarker Integration Network in Depression (CANBIND-1) data were tested on Establishing Moderators and Biosignatures of Antidepressant Response in Clinical Care (EMBARC) stage 1 sertraline, EMBARC stage 2 sertraline, and EMBARC stage 1 placebo data. Similarly, models trained on EMBARC stage 1 sertraline were tested on all other groups. Error bars represent 95% CIs (2.5%-97.5%), not adjusted for multiple comparisons. We compared dACC models with their respective clinical counterparts. Within-trial model performance was tested using repeated 10-fold cross-validation (CV). For dACC models, within-trial cross-validation was conducted on a full set of predictors; conversely, cross-trial bootstrapping was conducted on a smaller set of predictors (52 clinical and dACC variables) that survived regularization in CANBIND-1. Bootstrapping of the full predictor set can be found in eFigure 4 in Supplement 1. ^a^Denotes models whose performance was significantly higher than chance (1-tailed *P* < .05). ^b^Denotes significant differences between models (*P* < .10 from bootstrapping the differences in AUC).

AUC and balanced accuracy values for early treatment models are summarized in Table 2, and resampling results are shown in eFigure 3 and eFigure 4 in Supplement 1. We found that the early-treatment models performed the best overall, outperforming the clinical plus dACC pretreatment models. The clinical model reached out-of-trial AUCs of 0.66 to 0.73 and balanced accuracy of 66% to 69% when trained on CANBIND-1 and EMBARC data and tested on samples who received SSRIs rather than placebo (eTable in Supplement 1). Although the addition of dACC connectivity features improved the model fit in EMBARC stage 2, this improvement was not significant in bootstrapping analyses (Figure 3 and eFigure 5 in Supplement 1).

### Multivariate Regression Predicting Change in Depression Severity

A PLS-R model predicting change in depression severity in CANBIND-1 explained significantly more variance than expected by chance, whereas a similar PLS-R model explained significantly more variance than expected by chance in EMBARC stage 1. Models trained and tested on the same data showed very high levels of performance (eFigure 6 in Supplement 1); performance decreased when models were trained on one trial and tested on a different trial, with out-of-trial predicted vs observed correlations for SSRI-to-SSRI generalization ranging between 0.31 and 0.39. In sensitivity analyses, reanalyzing the data while applying batch harmonization (using ComBat) within trials reduced out-of-trial performance slightly, but did not alter the overall results (eAppendix 3 and eTable in Supplement 1).

## Discussion

The findings of this prognostic study identified promising biological and clinical markers of antidepressant treatment response. Overall cross-trial model prediction performance for SSRIs was encouraging, with baseline models including dACC connectivity features achieving cross-trial balanced accuracy of 63% to 71% in 2 multisite trials from Canada and the US. Importantly, early (2-week) depression severity produced model performance that was on par or better than performance of a combination of baseline clinical and fMRI FC features. Notably, neuroimaging predictors included lower connectivity of the dACC with dlPFC, which is consistent with transcranial magnetic stimulation (rTMS) studies for MDD.

Although our model performance was moderate, it was better than expected based on similar studies of schizophrenia treatment markers.^2^ This may be due to shared clinical features of the 2 trials considered here. Testing across trials featuring patient groups who vary widely in levels of severity, chronicity, and age groups^2^ may result in lower generalization. Differences in underlying cause likely underpin patient heterogeneity and may require context-dependent models that identify markers in more homogeneous patient populations (eg, treatment-resistant late-life depression vs nonresistant MDD in adults). We also harmonized clinical and neuroimaging data across trials, using the same fMRI processing streams and predictive features, and our results were relatively robust to batch harmonization. Nevertheless, the moderate performance likely reflects MDD heterogeneity.^28,29,30^

The circuit identified as predictive of treatment response included the anticorrelation between dACC and dlPFC as well as the angular gyrus. The dACC seed included posterior Brodmann area 24 and the anterior Brodmann area 32 prime. It is thus at the intersection between the hot and cold subdivisions of the dACC.^31^ A similar connectivity map has been previously identified as predicting treatment outcomes in CANBIND-1,^6^ despite differences in fMRI processing and statistical models. In addition, rTMS trials use the most anticorrelated portion of the dlPFC with the subgenual ACC as a target stimulation region to maximize effectiveness,^10,32^ implicating this circuit in both pharmacotherapy and rTMS response.^9^ These findings fit theories proposing that top-down emotional regulation is achieved by prefrontal regions regulating the ACC and amygdala activity, regions typically recruited by emotional stimuli.^33,34,35^

The inclusion of global cortical connectivity and gray matter structure did not improve prediction performance, consistent with previous studies of CANBIND-1 cortical thickness data.^36^ These biomarkers may be different in distinct patient populations, however. Finally, early treatment outcome data from week 2 depression severity scores were very informative as early improvements in depressive symptoms predicted treatment response at the end of a full course of treatment.

In addition to the main analyses of a binary response outcome variable, we also found substantial cross-trial generalizability of multivariate models predicting change in depression severity, with out-of-trial predicted vs observed correlations between 0.31 and 0.39. Overall, although our model performance is moderate, it shows that adding early treatment response information, as well as resting-state biomarkers, improves our ability to predict response after a full course of treatment. Integrating neuroimaging markers with other modalities, such as cognitive and psychological assessments, may further boost prediction performance.^3^

If the FC and clinical markers identified here are replicated, prospective trials with biomarker-guided treatment assignment will be needed to test the markers’ utility. Although using neuroimaging and cognitive markers to help assign treatments is promising, this research avenue is not without challenges, limited by the training data and generalizability of models as well as harmonization and similarity between different samples.^37^

### Limitations

Our study has some limitations. First, we included only 2 clinical trials, which limited our sample size. Lack of preregistration of the analytic approach is an additional limitation, although our methods follow previously published modeling approaches.^2^ Furthermore, data harmonization across trials can be challenging, because no approaches for prospective harmonization of data from new, previously unseen participants in novel biomarker-guided trials exist. Future work should combine data across trials for more robust biomarkers and reveal more mechanistic insights into treatment response. In addition, we focused on SSRIs, and future studies will be needed to develop robust biomarkers for different therapies, including pharmacological medications and rTMS.

## Conclusions

In conclusion, the current cross-trial generalization results represent an important step toward biomarkers of antidepressant response. Leveraging data to identify robust biomarkers that generalize across patient populations in different geographic locations will allow us to test such biomarkers in prospective randomized clinical trials and hopefully help connect patients with treatments that work best for them.

## Notes

Supplement 1. **eAppendix 1.** Demographic and clinical information **eFigure 1.** Overview of the participant flow in CANBIND and EMBARC **eAppendix 2.** Supplemental methods **eAppendix 3.** Supplemental results **eFigure 2.** Global functional connectivity (Global FC) predictors of treatment response in CANBIND-1 and EMBARC and the out-of-trial generalization performance **eFigure 3.** Receiver operating characteristic curves (A) and area-under the curve (AUC) histogram (B) for predicting response to sertraline and placebo in EMBARC after repeated 10-fold cross-validation training on CANBIND data for the clinical+dACC model **eTable.** Summary of out-of-trial model performance for models trained in the CANBIND and EMBARC clinical trials following ComBat batch harmonization **eFigure 4.** Area under the curve (AUC) for models predicting treatment response in clinical+dACC models with all 366 features derived from bootstrapping analyses **eFigure 5.** Difference in areas under the curve (ΔAUC) for dACC and clinical models predicting treatment response derived from bootstrapping analyses **eFigure 6.** Scatterplots of predicted vs observed change in HDRS-17 depression severity scores between the last clinical assessment and baseline
eReferences

Supplement 2.
Data Sharing Statement
