# Supplementary material for: Generalizability of Treatment Outcome Prediction Across Antidepressant Treatment Trials in Depression
Source: JAMA Netw Open. 2025 Mar 20;8(3):e251310. doi: 10.1001/jamanetworkopen.2025.1310 (PMC11926635; doi:10.1001/jamanetworkopen.2025.1310)
Supplement: Supplement 1. — eAppendix 1. Demographic and clinical information eFigure 1. Overview of the participant flow in CANBIND and EMBARC eAppendix 2. Supplemental methods eAppendix 3. Supplemental results eFigure 2. Global functional connectivity (Global FC) predictors of treatment response in CANBIND-1 and EMBARC and the out-of-trial generalization performance eFigure 3. Receiver operating characteristic curves (A) and area-under the curve (AUC) histogram (B) for predicting response to sertraline and placebo in EMBARC after repeated 10-fold cross-validation training on CANBIND data for the clinical+dACC model eTable. Summary of out-of-trial model performance for models trained in the CANBIND and EMBARC clinical trials following ComBat batch harmonization eFigure 4. Area under the curve (AUC) for models predicting treatment response in clinical+dACC models with all 366 features derived from bootstrapping analyses eFigure 5. Difference in areas under the curve (ΔAUC) for dACC and clinical models predicting treatment response derived from bootstrapping analyses eFigure 6. Scatterplots of predicted vs observed change in HDRS-17 depression severity scores between the last clinical assessment and baseline eReferences [file jamanetwopen-e251310-s001.pdf]

# Supplemental Online Content

Zhukovsky P, Trivedi MH, Weissman M, Parsey R, Kennedy S, Pizzagalli DA. Generalizability of treatment outcome prediction across antidepressant treatment trials in depression. *JAMA Netw Open*. 2025;8(3):e251310. doi:10.1001/jamanetworkopen.2025.1310

**eAppendix 1.** Demographic and clinical information

**eFigure 1.** Overview of the participant flow in CANBIND and EMBARC

**eAppendix 2.** Supplemental Methods

**eAppendix 3.** Supplemental Results

**eFigure 2.** Global functional connectivity (Global FC) predictors of treatment response in CANBIND-1 and EMBARC and the out-of-trial generalization performance

**eFigure 3.** Receiver operating characteristic curves (A) and area-under the curve (AUC) histogram (B) for predicting response to sertraline and placebo in EMBARC after repeated 10-fold cross-validation training on CANBIND data for the clinical+dACC model

**eTable.** Summary of out-of-trial model performance for models trained in the CANBIND and EMBARC clinical trials following ComBat batch harmonization

**eFigure 4.** Area under the curve (AUC) for models predicting treatment response in clinical+dACC models with all 366 features derived from bootstrapping analyses

**eFigure 5.** Difference in areas under the curve (ΔAUC) for dACC and clinical models predicting treatment response derived from bootstrapping analyses

**eFigure 6.** Scatterplots of predicted vs observed change in HDRS-17 depression severity scores between the last clinical assessment and baseline

**eReferences**

This supplemental material has been provided by the authors to give readers additional information about their work.

© 2025 Zhukovsky P et al. *JAMA Network Open*.

## eAppendix 1. Demographic and clinical information

We provide more details on the demographics of the Establishing Moderators and Biosignatures of Antidepressant Response in Clinical Care (EMBARC) and the Canadian Biomarker Integration Network in Depression (CANBIND) samples in Table 1. An overview of the participant flow is shown in Supplementary Figure 1. Employment status was determined as full or part-time employment in EMBARC; and ‘working now’ in CANBIND. The Body Mass Index (BMI) data in EMBARC included some extreme outliers, hence we excluded BMI data for those with BMI<13 and BMI>55. We used linear regression to impute BMI from waist circumference for those participants with available data. Pre-treatment depression severity was measured using the Montgomery Asberg Depression Rating Scale (MADRS)[1] in CANBIND and Hamilton Depression Rating Scale (HDRS) [2] in EMBARC. We converted MADRS scores to HDRS-17 scores as part of data harmonization using a previously described approach[3] (see also https://mood-disorders.co.uk/ASSETS/FILES/QIDS-MADRS-HRSD-conversion-table-pdf.pdf). The conversion approach used a previously published table mapping between HDRS-17 and MADRS scores. This approach has been extensively validated using item response theory analyses[3]. While the mapping is mostly linear, sometimes a range of scores from one questionnaire corresponds to one score on the other questionnaire. For instance, a MADRS score of 8 or 9 corresponds to an HDRS-17 score of 7 while a MADRS score of 10 corresponds to a HDRS-17 score of 8. We provide the code used for this mapping in a public github repository:
https://github.com/peterzhukovsky/MDD_response_prediction/blob/main/madrs2hdrs17.m

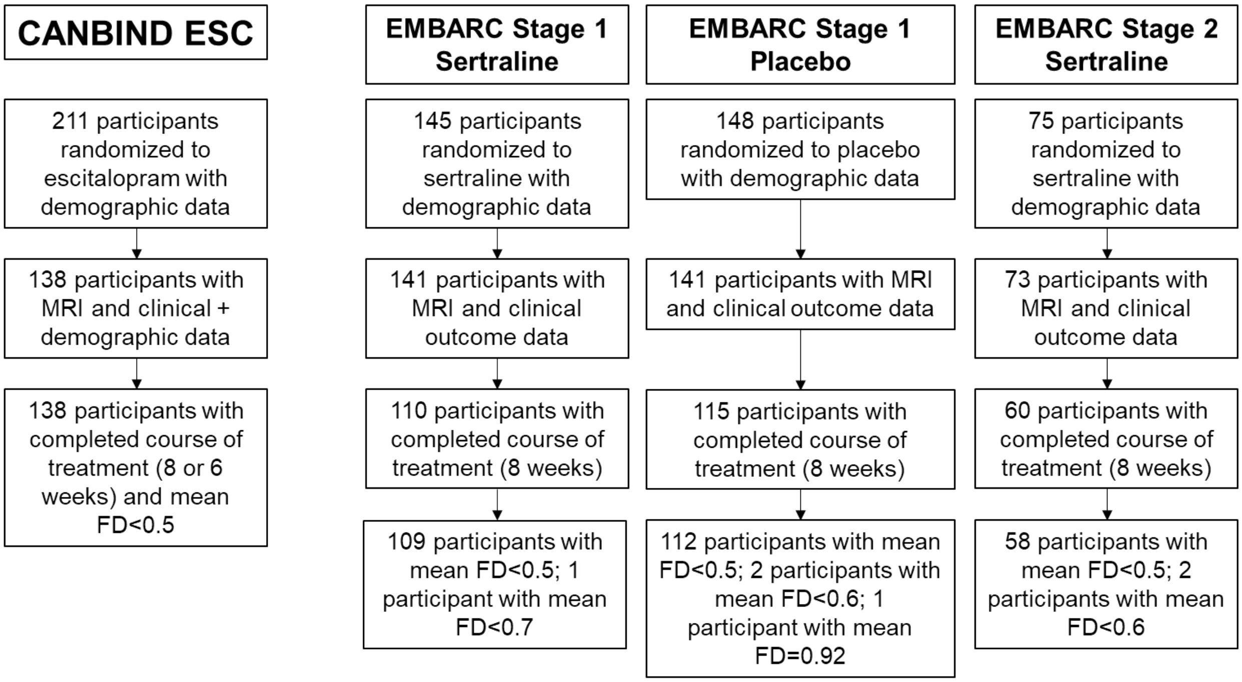


**eFigure 1.** Overview of the participant flow in CANBIND and EMBARC. MRI data refer to baseline MRI only.

© 2025 Zhukovsky P et al. *JAMA Network Open*.

## eAppendix 2. Supplemental Methods

*Participants*. EMBARC included 296 outpatients 18–65 years of age recruited from four academic sites in the US (Columbia University, Massachusetts General Hospital, University of Michigan, UT Southwestern Medical Center) who were diagnosed as having recurrent or chronic MDD and were not taking medication for MDD; participants were not required to be medication naive. They took part in an MRI session that included both resting state fMRI and structural MRI imaging. To test our hypotheses, we defined three population subgroups, with the first group being treated with sertraline in Stage 1 (n=110 with complete data), the second group being treated with sertraline in Stage 2 after not responding to placebo in Stage 1 (i.e., a different set of n=64) and the third group receiving placebo in Stage 1 (n=115). CANBIND included 144 MDD patients 18-61 years of age recruited from six sites in Canada (Toronto General Hospital (TGH); CAMH (CAM); McMaster University (MCU);University of Calgary (UCA); University of British Columbia (UBC); Queens University (QNS)) who completed a resting state fMRI and structural MRI scan before starting escitalopram treatment. Among those participants, 138 had completed at least six weeks of escitalopram treatment and had complete baseline prediction data.

*Clinical data.* Participants provided information on their age, sex, employment status (binarized as unemployed vs. partially or fully employed here) and overlapping clinical data were available on baseline depression severity (MADRS [1] in CANBIND and 17-item HDRS [2] in EMBARC) and anhedonia (Snaith Hamilton Rating Scale (SHAPS) [4]). BMI scores were also available in both datasets. BMI outlier scores (<15 or >55) were removed in EMBARC; we imputed BMI using regression of waist circumference scores for participants who had those data available. No outliers were present in CANBIND. Finally, to harmonize across prediction models, we converted MADRS to HRSD scores in CANBIND [3].

*MRI Data.* We preprocessed structural and resting-state fMRI data in EMBARC using fMRIPrep v22.1.1 and in CANBIND using fMRIPrep v23.0.2[5]. For denoising, we regressed out 24 fixed confounds: 6 motion parameters, average signal from the white matter and cerebrospinal fluid and their first and second-order temporal derivatives. Next, we applied a 6-mm smoothing kernel. We registered fMRIPrep outputs from the MNI152 (Nlin6) space to the FreeSurfer fsaverage space (mri_vol2surf) and extracted fMRI timeseries for 360 cortical regions in the Human Connectome Project (HCP) parcellation [6]. We then calculated global cortical FC measures by averaging the rows of a 360 x 360 connectivity matrix, excluding the values along the diagonal. In addition to the global cortical FC measures, due to a priori hypotheses, we also calculated seed-based FC of the dorsal anterior cingulate (dACC) and rostral anterior cingulate (rACC), respectively. Seeds were selected based on the bilateral dACC and rACC regions that survived regularization in predicting treatment response in CANBIND. The dACC seed comprised bilateral p24 and a32pr regions, while the rACC seed comprised bilateral a24 and p32 regions from the HCP parcellation (Figure 1B, 1D). Structural data were processed using FreeSurfer as part of the fMRIPrep pipeline, producing cortical thickness outputs in the aparc parcellation[7], and subcortical volumes in the aseg parcellation. Subcortical volumes were divided by the total intracranial volumes to provide normalized values.

While EMBARC also includes arterial spin labeling data [8,9] and both EMBARC [10,11] and CANBIND [12,13] have task-based fMRI with different tasks (e.g., emotion conflict monitoring in EMBARC and Go/NoGo and incentive delay in CANBIND), we focus on imaging data that are common across both EMBARC and CANBIND, namely structural MRI and resting-state functional connectivity. Future studies should also examine other imaging modalities such (e.g. ASL) as candidate biomarkers of treatment response.

*Predictive modeling approach*. We used elastic net logistic regressions with regularization (*lassoglm*, Matlab R2022a [16]) to predict treatment outcomes in the four datasets. While various machine learning approaches with non-linear prediction exist, recent evidence from large-scale brain-behavior studies suggests that linear models perform on par with some of the more complex prediction models [17]. Given the

© 2025 Zhukovsky P et al. *JAMA Network Open*.

moderate size of the imaging datasets analyzed here, and following recent studies of treatment outcome prediction [18], we use elastic net logistic models aiming to maintain a higher feature-to-observation ratio [19]. Regularization also allows us to identify a smaller, most salient set of predictors that could be tested in future studies. There were five sets of models tested using baseline depression severity: (1) a clinical model including age, sex, employment, baseline HDRS, SHAPS, and BMI; (2) a clinical + global FC model that included all features from the clinical model and added 360 global FC features; (3) a clinical + dACC FC model that added 360 seed-based dACC features to the clinical model; (4) a clinical + rACC FC model that added 360 seed-based rACC features to the clinical model; and (5) a clinical + cortical thickness (CT) model that added 74 gray matter features to the clinical model. We then tested five analogous models that included week 2 instead of baseline depression severity scores. We provide an overview of the models below:

(1) response ~ age + sex + employment + baseline HDRS + SHAPS + BMI
(2) response ~ age + sex + employment + baseline HDRS + SHAPS + BMI + global FC
(3) response ~ age + sex + employment + baseline HDRS + SHAPS + BMI + dACC FC
(4) response ~ age + sex + employment + baseline HDRS + SHAPS + BMI + rACC FC
(5) response ~ age + sex + employment + baseline HDRS + SHAPS + BMI + brain structure

First, we tested the prediction performance of models within CANBIND and EMBARC Stage 1 by creating 100 random training and test data splits, training the elastic net model with 10-fold cross-validation on the training dataset, and predicting outcomes in the test dataset on each iteration. Second, we evaluated the prediction performance of models trained on CANBIND and tested in EMBARC Stage 1, EMBARC Stage 2, and EMBARC placebo. Training models used 10-fold cross-validation to optimize regression weights. This process resulted in point-estimates of AUC and balanced accuracy for out-of-trial prediction performance, reported in Table 2. Balanced accuracy was calculated as the mean of sensitivity and specificity [18].

We next used bootstrapping (bootfun in Matlab) to assess whether AUCs were significantly higher than chance and to compare the most promising models with each other. We conducted two sets of bootstrapping analyses: first, across 1,000 bootstraps, we sampled from CANBIND and tested the out-of-trial performance of models with clinical features only and those with clinical and dACC FC features that survive regularization in the EMBARC datasets. Second, we sampled from EMBARC Stage 1 and tested the out-of-sample performance of models with clinical features only and those with clinical and dACC FC features that survive regularization (n=1,000 iterations) in CANBIND and in EMBARC Stage 2 and EMBARC placebo data. A limitation of this approach is that while the models are trained on EMBARC Stage 1 data and tested on CANBIND and EMBARC Stage 2 data, they only include features that survive regularization in original CANBIND training. While this process makes feature selection circular, feature tuning or coefficient selection is not. Models were deemed to perform significantly higher than chance by comparing the AUCs from the bootstrap distribution to AUC=0.5. We calculated the p-values by dividing the number of bootstraps with AUC below 0.5 by 1,000 with a one-tailed p-value threshold of 0.05 for significance (50 out of 1,000 iterations). When comparing between models, we calculated the difference in AUC for pairs of models (i.e., clinical models vs. clinical and dACC-to-cortex FC models) on each bootstrap and calculated the p-values by dividing the number of bootstraps with $\Delta$AUC<0 by 1,000, with a one-tailed p-value threshold of 0.05. We removed models leaving no variables after regularization, primarily among clinical-only models, affecting up to 10% of bootstraps. In those cases, following previous studies [18], we did not include the AUC in our plots and analyses. In our elastic net models, we used the hyperparameter $\alpha$=0.01 for models trained on CANBIND data and $\alpha$=0.001 for models trained on EMBARC data. One-tailed p-value thresholds were chosen given that we tested whether models performed significantly better than chance and whether more complex models including connectivity features performed better than clinical models.

© 2025 Zhukovsky P et al. *JAMA Network Open*.

*Hyperparameter optimization.* The elastic net models used the default hyperparameter optimization options (*lassoglm.m*). We used the following alpha hyperparameters: α=0.3 for clinical+fMRI models trained in CANBIND; α=0.1 for structural MRI models trained in CANBIND; and α=0.001 for models trained on clinical models or models trained on EMBARC data.

We tested a range of α thresholds when training the model within the CANBIND or EMBARC data α=[0.001, 0.01, 0.1, 0.2, 0.3, 0.4, 0.5, 0.6], and selected the above values as they produced a reasonable set of predictors at the training stage. Models with higher α criteria resulted in more conservative regularization and often did not return any predictors surviving regularization. Conversely, models with very low α criteria returned regression weights for nearly every predictor variable. Since we expected only some imaging features to be contributing to predictions, we selected α criteria that would prune over 50% of the functional connectivity features or brain structure features. We also reran the models with the best performing α values several times and selected the α value for which the model returned the nearly same number of features every time.

When reporting balanced accuracy values, we preferentially select balanced accuracy values based on both sensitivity and specificity being larger then 0.55; if that was not possible, we selected balanced accuracy values based on both sensitivity and specificity being larger then 0.50. Sometimes, higher balanced accuracy is possible at the trade-off cost of having either low sensitivity and high specificity or vice versa; however, we were aiming to balance both sensitivity and specificity performance.

*Data harmonization*. We repeated the analyses described above twice. First, we report findings without batch harmonization. Current batch harmonization tools require full datasets to estimate site- and confound-specific biases, posing the challenge of prospective harmonization to a new patient from a new site. To test generalizability in context of potential clinical trials, we wanted to test model performance with the currently available tools. However, we also report results of predicting modelling after batch harmonization in ComBat (see Supplementary Section 3.2).

*Sensitivity analyses.* First, we reanalyzed the data while correcting for batch effects in the rs-fMRI data and the gray matter brain structure using ComBat [20] https://github.com/Jfortin1/ComBatHarmonization/ in the Supplementary Information.

To test the robustness of our findings we conducted several sensitivity analyses. First, while we report in the main results the bootstrapped performance of the most parsimonious model featuring 52 predictors, we also bootstrapped elastic net models with the full set of 366 predictors. This analysis allowed the elastic net models to prune any predictors via regularization on each bootstrap iteration in addition to fitting regression weights to a specific set of predictors. We found a similar bootstrapping performance in this analysis to that of the 52-predictor model (Supplementary Section 3.3).

*Multivariate regression predicting change in depression severity.* In this secondary analysis, we aimed to test model performance predicting change in HDRS-17 scores in EMBARC and the MADRS scores transformed to HDRS-17 scores in CANBIND. Absolute difference between 8-week and baseline was used as an outcome. Whenever 8-week outcomes were not available, 6-week scores were used in CANBIND. In EMBARC Stage 2, 12-week scores were available while in EMBARC Stage 1, 6-week scores were available. Given that a 4-week course of treatment is short, and we aimed for consistency within each trial we only include EMBARC data with a full 8-week course of treatment. Partial least squares models with 360 dACC connectivity features, age, sex, employment, baseline HDRS-17, SHAPS, and BMI were run. These were the same predictors as those used in the main analyses. We used permutation testing (n=5,000) and bootstrapping (n=5,000, $Z > 3$ and $Z < -3$) to assess model significance and to identify robust features. We trained the model on CANBIND and then applied the regression weights from the resulting model to predict change in depression severity in EMBARC; similarly, we trained the model on EMBARC

© 2025 Zhukovsky P et al. *JAMA Network Open*.

Stage 1 sertraline and applied the regression weights to predict change in depression scores in the other datasets.

## eAppendix 3. Supplemental Results

### 3.1. Global functional connectivity maps predicting treatment response

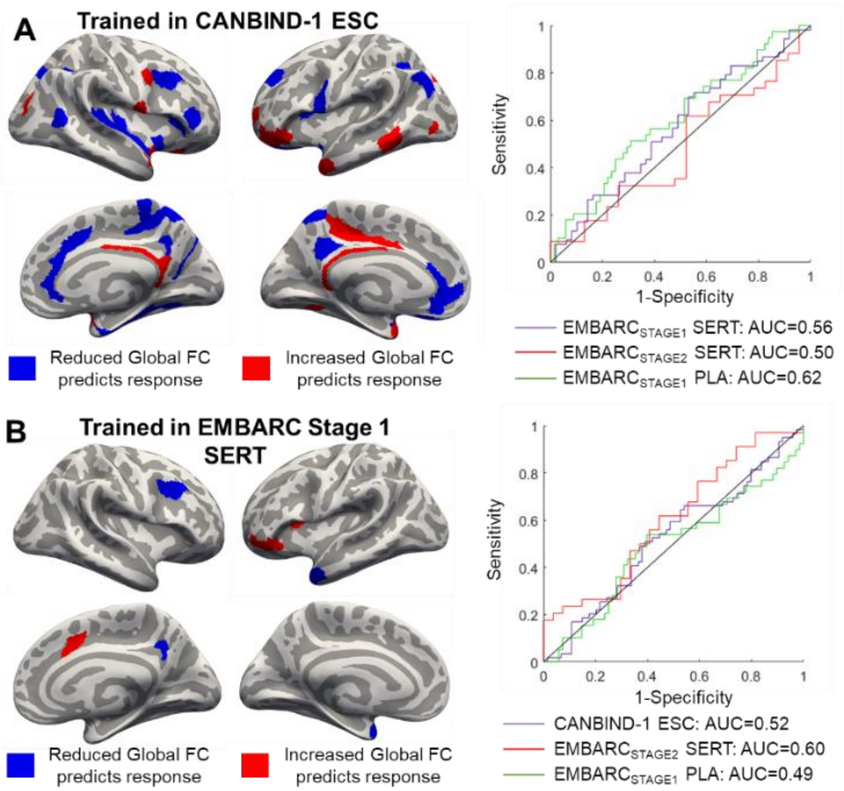


**eFigure 2.** Global functional connectivity (Global FC) predictors of treatment response in CANBIND-1 and EMBARC and the out-of-trial generalization performance. We first trained models on CANBIND-1 escitalopram data **(A)** and then tested them on EMBARC Stage 1 sertraline, EMBARC Stage 2 sertraline, and EMBARC Stage 1 placebo groups. We then trained models on the EMBARC Stage 1 sertraline sample **(B)** and tested the resulting models on CANBIND-1 escitalopram, EMBARC Stage 2 sertraline and EMBARC Stage 1 placebo samples. We show the global FC maps predicting response in CANBIND-1 **(A)** and EMBARC **(B)** alongside the respective out-of-trial receiver-operator curve (ROC-AUC) analyses. ESC: escitalopram, FC: functional connectivity, SERT: sertraline, AUC: area under the curve.

### 3.2. Model stability

We evaluated the stability of models following repeated training. Training the regularized elastic net models involved random data splits, which may produce slightly different models on each iteration, and we wanted to test whether model performance for the clinical+dACC (dorsal Anterior Cingulate Cortex) varied depending on the training split. We found the best performing pre-treatment model (including dACC connectivity features) to be very stable (Supplementary Figure S3).

© 2025 Zhukovsky P et al. *JAMA Network Open*.

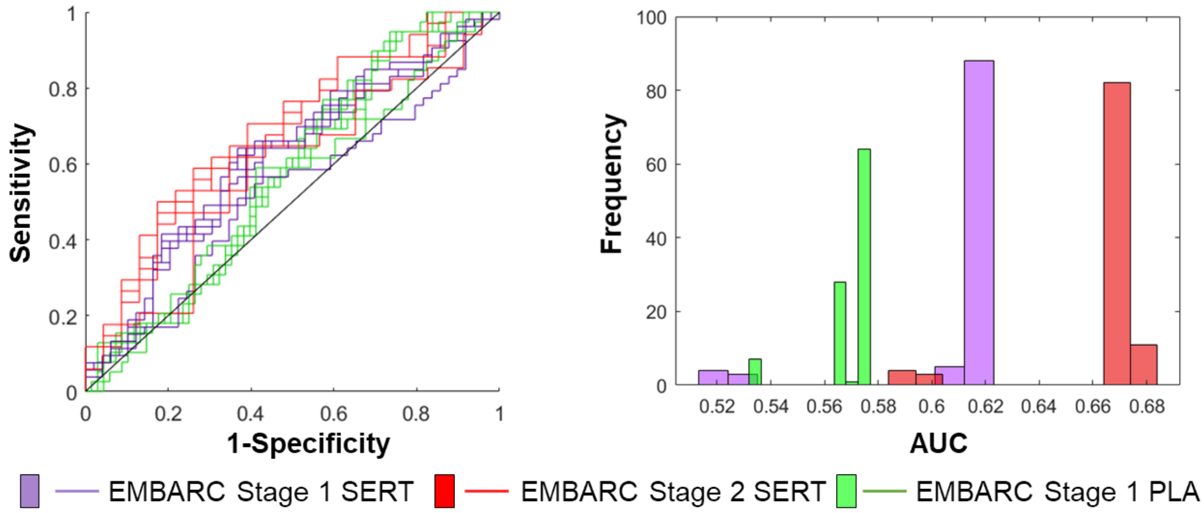


**eFigure 3.** Receiver operating characteristic curves **(A)** and area-under the curve (AUC) histogram **(B)** for predicting response to sertraline and placebo in EMBARC after repeated 10-fold cross-validation training on CANBIND data for the clinical+dACC model. Models trained on CANBIND data were tested on EMBARC Stage 1 sertraline, EMBARC Stage 2 sertraline and EMBARC Stage 1 placebo data. We plot receiver-operator curves after running the same model 100 times. On every iteration, the model was trained using a different 10-fold data split, with regularization leading to slightly different model coefficients. Regularization hyperparameter was set at $\alpha$=0.3 for this fMRI model. Overall, the AUCs are very similar across the iterations. SERT: sertraline; PLA: Placebo

### 3.3. Model performance following batch harmonization using ComBat

We applied ComBat batch harmonization to adjust for age, sex and scanning site within each trial. We then repeated the main out-of-trial prediction analyses from the main text. We found the results of this re-analysis (shown in Supplementary Table 2) to be largely consistent with the findings reported in the Table 1 of the main text. The overall out-of-trial model performance was similar, and the addition of dACC connectivity features also improved model performance in this analysis.

© 2025 Zhukovsky P et al. *JAMA Network Open*.

| | Models trained on CANBIND ESC | | | | | | Models trained on EMBARC Stage 1 SERT | | | | | |
|---|---|---|---|---|---|---|---|---|---|---|---|---|
| **Pre-treatment models of response** | | | | | | | | | | | | |
| **Tested on:** | **EMBARC Stage 1 SERT** | | **EMBARC Stage 2 SERT** | | **EMBARC Stage 1 PLA** | | **CANBIND Escitalopram** | | **EMBARC Stage 2 SERT** | | **EMBARC Stage 1 PLA** | |
| | **AUC** | **bACC** | **AUC** | **bACC** | **AUC** | **bACC** | **AUC** | **bACC** | **AUC** | **bACC** | **AUC** | **bACC** |
| Clinical Model* | 0.58 | **0.61** | 0.58 | **0.60** | 0.63 | **0.59** | 0.59 | **0.59** | 0.61 | **0.60** | 0.52 | 0.55 |
| Clinical + Global FC# | 0.55 | 0.57 | 0.49 | 0.52 | 0.63 | **0.63** | 0.45 | 0.46 | 0.60 | 0.55 | 0.49 | 0.49 |
| **Clinical + dACC FC#** | 0.61 | **0.61** | 0.64 | **0.62** | 0.55 | 0.56 | 0.71 | **0.68** | 0.67 | **0.66** | 0.61 | **0.63** |
| Clinical + rACC FC# | 0.61 | **0.64** | 0.66 | **0.67** | 0.66 | **0.62** | 0.44 | 0.51 | 0.49 | 0.51 | 0.44 | 0.47 |
| Clinical + CT^ | 0.60 | 0.57 | 0.63 | 0.56 | 0.64 | **0.61** | 0.60 | **0.63** | 0.65 | **0.68** | 0.51 | 0.56 |
| ***Age, Sex, SHAPS, Employment, BMI, baseline HDRS/MADRS** | | | | | | | | | | | | |
| **Early treatment models of response** | | | | | | | **Early treatment models of response** | | | | | |
| **Clinical Model*** | 0.68 | **0.69** | 0.73 | **0.66** | 0.71 | **0.66** | 0.66 | **0.69** | 0.68 | **0.66** | 0.63 | **0.66** |
| Clinical + Global FC# | 0.64 | **0.67** | 0.63 | **0.60** | 0.70 | **0.65** | no variables survive regularization | | | | | |
| Clinical + dACC FC# | 0.67 | **0.62** | 0.78 | **0.74** | 0.69 | **0.65** | 0.67 | **0.62** | 0.74 | **0.74** | 0.64 | **0.65** |
| Clinical + rACC FC# | 0.66 | **0.64** | 0.77 | **0.73** | 0.74 | **0.68** | no variables survive regularization | | | | | |
| Clinical + CT^ | 0.66 | **0.63** | 0.80 | **0.77** | 0.72 | **0.65** | 0.56 | 0.56 | 0.68 | **0.63** | 0.55 | 0.56 |

***Age, Sex, SHAPS, Employment, BMI, baseline HDRS/MADRS, change in HDRS/MADRS at week 2**

#FC models trained on EMBARC1 used variables derived from the CANBIND models

^CT models trained on EMBARC1 used variables derived from the CANBIND models

**eTable.** Summary of out-of-trial model performance for models trained in the CANBIND and EMBARC clinical trials following ComBat batch harmonization. AUC: area under the curve; bACC: balanced accuracy. Balanced accuracy values highlighted in bold were significantly higher than chance (p<0.05, not correcting for the number of models) based on prior simulations [33].

## 3.4. Bootstrapping the full set of dACC predictors

We re-ran the bootstrapping of the out-of-trial model performance in CANBIND using all potential 366 predictors, which allowed us to apply regularization ($\alpha$=0.3) and fit regression weights for the surviving predictors on each bootstrap iteration. We show the results of this re-analysis in Supplementary Figure S4. Overall, we found bootstrapping performance in this analysis to be similar to the performance of the 52-predictor model reported in the main analyses.

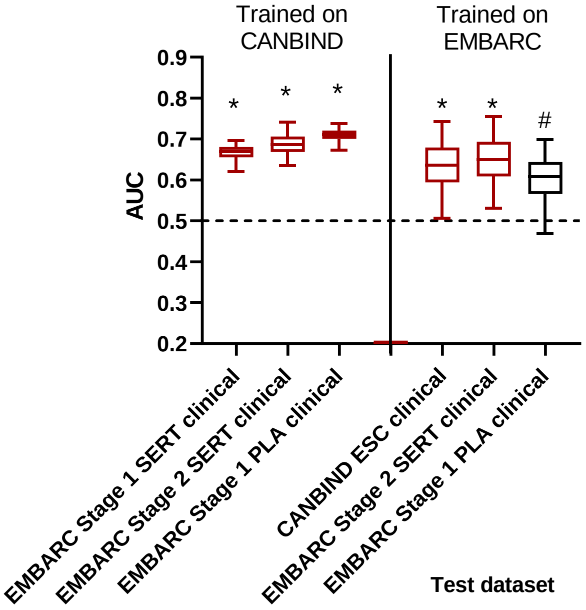


**eFigure 4.** Area under the curve (AUC) for models predicting treatment response in clinical+dACC models with all 366 features derived from bootstrapping analyses. Models trained on CANBIND data were tested on EMBARC Stage 1 sertraline (EMBARC1), EMBARC Stage 2 sertraline (EMBARC2) and EMBARC Stage 1 placebo data (EMBARC1 PLA). Error bars represent 95% confidence intervals [2.5%-97.5%], not adjusted for multiple comparisons. Boxplots of models whose performance was significantly higher than chance (one-tailed *p<0.05, #p<0.1) are highlighted in red. We compared dACC models with their respective clinical counterparts, with significant differences between models highlighted with a bar (one-tailed *p<0.05; #p<0.1 from bootstrapping the differences in AUC).

© 2025 Zhukovsky P et al. *JAMA Network Open*.

### 3.5. Model comparison

The model performance was improved by the addition of dACC features when the models were trained on CANBIND and tested on EMBARC Stage 1 SERT (p=0.0824) and on EMBARC Stage 2 SERT (p=0.033); model performance was also improved by the addition of dACC features when the models were trained on EMBARC Stage 1 SERT and tested on EMBARC Stage 1 PLA (p=0.068) as shown in Supplementary Figure S5.

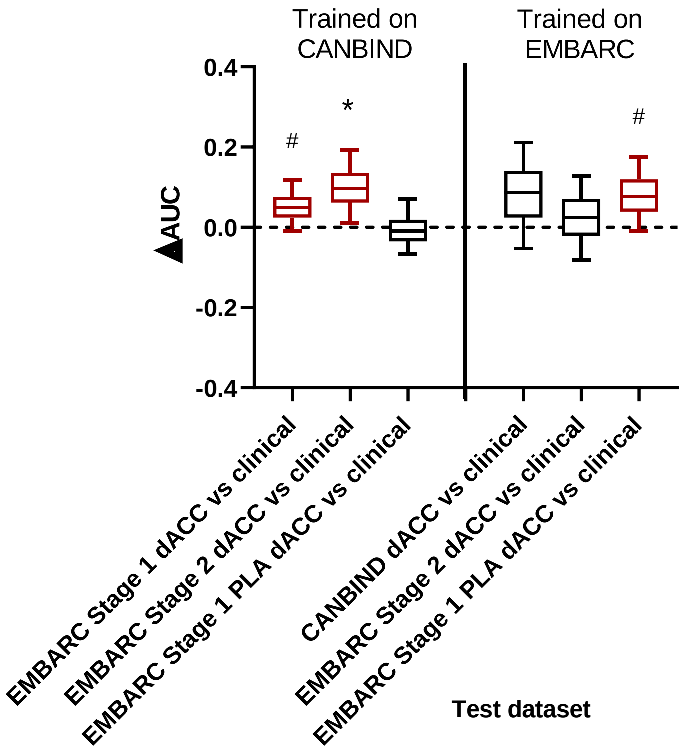


**eFigure 5.** Difference in areas under the curve (ΔAUC) for dACC and clinical models predicting treatment response derived from bootstrapping analyses. Models trained on CANBIND data were tested on EMBARC Stage 1 sertraline, EMBARC Stage 2 sertraline and EMBARC Stage 1 placebo data. Similarly, models trained on EMBARC Stage 1 sertraline were tested on all other groups. dACC models included 52 features in total. Error bars represent 95% confidence intervals [2.5%-97.5%], not adjusted for multiple comparisons. Boxplots of dACC models that performed better than the clinical models across bootstraps (one-tailed *p<0.05; #p<0.1) are highlighted in red.

© 2025 Zhukovsky P et al. *JAMA Network Open*.

### 3.6. Partial Least Squares Regression predicting change in depression severity

We found the model predicting change in depression severity in CANBIND to explain significantly more variance than expected by chance (permutation p=0.004), with various dACC functional connectivity features and baseline depression severity crossing the Z > 3 or Z < -3 threshold after bootstrapping. Similarly, we found the model predicting change in depression severity in EMBARC Stage 1 to explain significantly more variance than expected by chance (permutation p=0.0298), with various dACC functional connectivity features and baseline depression severity crossing the Z > 3 or Z < -3 threshold after bootstrapping. Similar to the main analyses predicting treatment response, we found that models trained and tested on the same data showed very high levels of performance. However, performance decreased when models were trained on one trial and tested on a different trial, with out of trial predicted vs observed correlations for SSRI-to-SSRI generalization ranging between 0.3 and 0.4. Models trained on SSRI data and used to predict changes in depression severity to placebo showed predicted vs observed correlations of approximately 0.2. We show all predicted vs. observed correlations in Supplementary Figure S6.

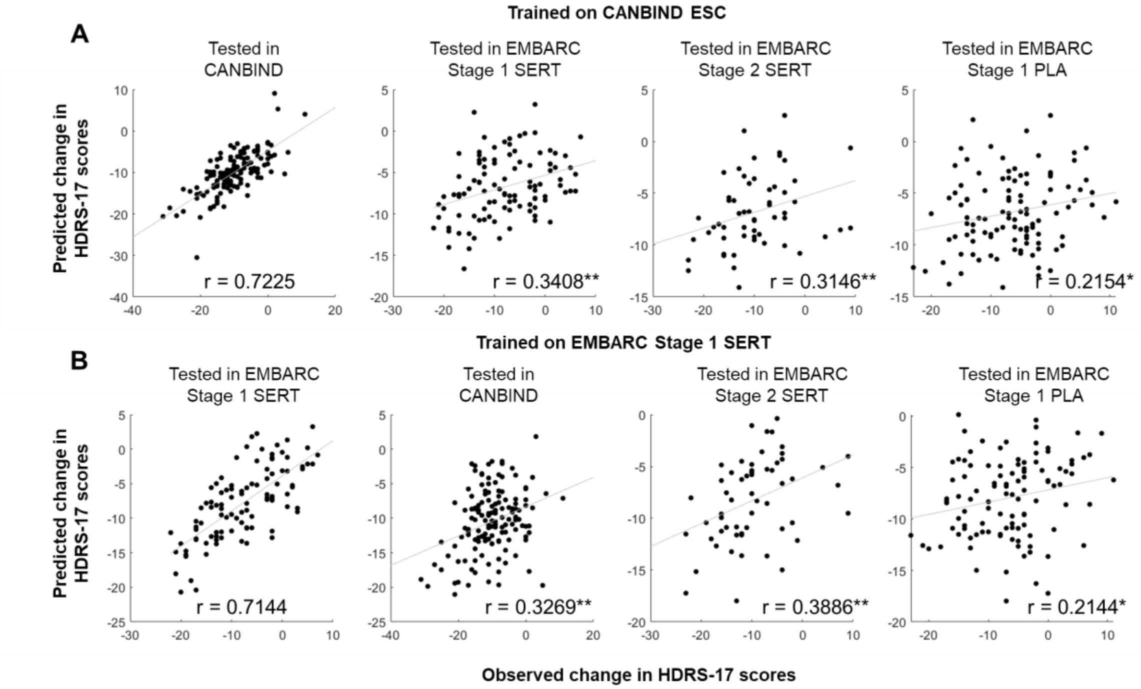


**eFigure 6.** Scatterplots of predicted vs observed change in HDRS-17 depression severity scores between the last clinical assessment and baseline. Partial least squares regression models were first trained on CANBIND data and tested within CANBIND, on EMBARC Stage 1 sertraline, Stage 2 sertraline and Stage 1 placebo (A). Similarly, models were trained on EMBAR Stage 1 sertraline data and tested within EMBARC Stage 1 sertraline, on CANBIND, on Stage 2 sertraline and Stage 1 placebo (B). Pearson's correlations (r) for observed vs predicted values are shown. Uncorrected p<0.05*; uncorrected p<0.01**.

© 2025 Zhukovsky P et al. *JAMA Network Open*.

## eReferences


1. Montgomery A, Asberg M. A New Depression Scale Designed to be Sensitive to Change. *Br J Psychiatry*. 1979;134:382-389.

2. Hamilton M. Development of a rating scale for depressive illness. *Br J Soc Clin Psychol*. 1967;6:278-296. doi:10.1159/000395073

3. Carmody TJ, Rush AJ, Bernstein I, et al. The Montgomery Äsberg and the Hamilton ratings of depression: A comparison of measures. *Eur Neuropsychopharmacol*. 2006;16(8):601-611. doi:10.1016/j.euroneuro.2006.04.008

4. Snaith RP, Hamilton M, Morley S, Humayan A, Hargreaves D, Trigwell P. A scale for the assessment of hedonic tone. The Snaith-Hamilton Pleasure Scale. *Br J Psychiatry*. 1995;167(JULY):99-103. doi:10.1192/bjp.167.1.99

5. Esteban O, Markiewicz CJ, Blair RW, et al. fMRIPrep: a robust preprocessing pipeline for functional MRI. *Nat Methods*. 2019;16(1):111-116. doi:10.1038/s41592-018-0235-4

6. Glasser MF, Coalson TS, Robinson EC, et al. A multi-modal parcellation of human cerebral cortex. *Nature*. 2016;536(7615):171-178. doi:10.1038/nature18933

7. Desikan RS, Ségonne F, Fischl B, et al. An automated labeling system for subdividing the human cerebral cortex on MRI scans into gyral based regions of interest. *Neuroimage*. 2006;31(3):968-980. doi:10.1016/j.neuroimage.2006.01.021

8. Poirot MG, Ruhe HG, Mutsaerts HJMM, et al. Treatment Response Prediction in Major Depressive Disorder Using Multimodal MRI and Clinical Data: Secondary Analysis of a Randomized Clinical Trial. *Am J Psychiatry*. 2024;181(3):223-233. doi:10.1176/appi.ajp.20230206

9. Cooper CM, Chin Fatt CR, Jha M, et al. Cerebral Blood Perfusion Predicts Response to Sertraline versus Placebo for Major Depressive Disorder in the EMBARC Trial. *EClinicalMedicine*. 2019;10(April):32-41. doi:10.1016/j.eclinm.2019.04.007

10. Nguyen KP, Chin Fatt C, Treacher A, et al. Patterns of Pretreatment Reward Task Brain Activation Predict Individual Antidepressant Response: Key Results From the EMBARC Randomized Clinical Trial. *Biol Psychiatry*. 2022;91(6):550-560. doi:10.1016/j.biopsych.2021.09.011

11. Chase HW, Fournier JC, Greenberg T, et al. Accounting for dynamic fluctuations across time when examining fMRI test-retest reliability: Analysis of a reward paradigm in the EMBARC study. *PLoS One*. 2015;10(5):1-20. doi:10.1371/journal.pone.0126326

12. Macqueen GM, Hassel S, Arnott SR, et al. The canadian biomarker integration network in depression (CAN-BIND): Magnetic resonance imaging protocols. *J Psychiatry Neurosci*. 2019;44(4):223-236. doi:10.1503/jpn.180036

13. Alders GL, Davis AD, MacQueen G, et al. Reduced accuracy accompanied by reduced neural activity during the performance of an emotional conflict task by unmedicated patients with major depression: A CAN-BIND fMRI study. *J Affect Disord*. 2019;257(April):765-773. doi:10.1016/j.jad.2019.07.037

14. Markello RD, Arnatkevičiūtė A, Poline JB, Fulcher BD, Fornito A, Misic B. Standardizing workflows in imaging transcriptomics with the Abagen toolbox. *Elife*. 2021;10:1-27. doi:10.7554/eLife.72129

15. Hansen JY, Shafiei G, Markello RD, et al. Mapping neurotransmitter systems to the structural and functional organization of the human neocortex. *Nat Neurosci*. 2022;25(November):1569–1581. doi:10.1038/s41593-022-01186-3

16. Zou H, Hastie T. Regularization and variable selection via the elastic net. *J R Stat Soc Ser B Stat Methodol*. 2005;67(2):301-320. doi:10.1111/j.1467-9868.2005.00503.x


© 2025 Zhukovsky P et al. *JAMA Network Open*.

17. Schulz MA, Yeo BTT, Vogelstein JT, et al. Different scaling of linear models and deep learning in UKBiobank brain images versus machine-learning datasets. *Nat Commun*. 2020;11(1). doi:10.1038/s41467-020-18037-z

18. Chekroud AM, Hawrilenko M, Loho H, et al. Illusory generalizability of clinical prediction models. *Science (80- )*. 2024;383:164-167. doi:10.1126/science.adg8538

19. Helmer M, Warrington S, Mohammadi-Nejad AR, et al. On the stability of canonical correlation analysis and partial least squares with application to brain-behavior associations. *Commun Biol*. 2024;7(1). doi:10.1038/s42003-024-05869-4

20. Johnson WE, Li C. Adjusting batch effects in microarray expression data using empirical Bayes methods. Published online 2007:118-127. doi:10.1093/biostatistics/kxj037

21. Morgan SE, Seidlitz J, Whitaker KJ, et al. Cortical patterning of abnormal morphometric similarity in psychosis is associated with brain expression of schizophrenia-related genes. *Proc Natl Acad Sci*. Published online 2019:201820754. doi:10.1073/pnas.1820754116

22. Zhukovsky P, Wainberg M, Milic M, et al. Multiscale neural signatures of major depressive, anxiety, and stress-related disorders. *Proc Natl Acad Sci*. 2022;119(23):1-10. doi:10.1073/pnas.2204433119

23. Zhukovsky P, Savulich G, Morgan S, Dalley JW, Williams GB, Ersche KD. Morphometric similarity deviations in stimulant use disorder point towards abnormal brain ageing. *Brain Commun*. 2022;4(3). doi:10.1093/braincomms/fcac079

24. Morgan SE, Seidlitz J, Whitaker KJ, et al. Cortical patterning of abnormal morphometric similarity in psychosis is associated with brain expression of schizophrenia-related genes. *Proc Natl Acad Sci*. 2019;116(19):9604-9609. doi:10.1073/pnas.1820754116

25. French L, Ma T, Oh H, Tseng GC, Sibille E. Age-Related Gene Expression in the Frontal Cortex Suggests Synaptic Function Changes in Specific Inhibitory Neuron Subtypes. 2017;9(May):1-14. doi:10.3389/fnagi.2017.00162

26. Zhukovsky P, Ironside M, Duda JM, et al. Acute stress increases striatal connectivity with cortical regions enriched for μ- and κ-opioid receptors. *Biol Psychiatry*. 2024;(14):1-10. doi:10.1016/j.biopsych.2024.02.005

27. Howard DM, Adams MJ, Shirali M, et al. Genome-wide association study of depression phenotypes in UK Biobank identifies variants in excitatory synaptic pathways. *Nat Commun*. 2018;9(1):1-10. doi:10.1038/s41467-018-03819-3

28. Anderson KM, Collins MA, Kong R, et al. Convergent molecular, cellular, and cortical neuroimaging signatures of major depressive disorder. *Proc Natl Acad Sci*. 2020;117(40):202008004. doi:10.1073/pnas.2008004117

29. Fabbri C, Corponi F, Souery D, et al. The Genetics of Treatment-Resistant Depression: A Critical Review and Future Perspectives. *Int J Neuropsychopharmacol*. 2019;22(2):93-104. doi:10.1093/ijnp/pyy024

30. Wigmore EM, Hafferty JD, Hall LS, et al. Genome-wide association study of antidepressant treatment resistance in a population-based cohort using health service prescription data and meta-analysis with GENDEP. *Pharmacogenomics J*. 2020;20(2):329-341. doi:10.1038/s41397-019-0067-3

31. Li QS, Tian C, Seabrook GR, Drevets WC, Narayan VA. Analysis of 23andMe antidepressant efficacy survey data: implication of circadian rhythm and neuroplasticity in bupropion response. *Transl Psychiatry*. 2016;6(9):1-9. doi:10.1038/TP.2016.171

32. Fabbri C, Hagenaars SP, John C, et al. Genetic and clinical characteristics of treatment-resistant

© 2025 Zhukovsky P et al. *JAMA Network Open*.

depression using primary care records in two UK cohorts. *Mol Psychiatry*. 2021;26(7):3363-3373. doi:10.1038/s41380-021-01062-9

33. Combrisson E, Jerbi K. Exceeding chance level by chance: The caveat of theoretical chance levels in brain signal classification and statistical assessment of decoding accuracy. *J Neurosci Methods*. 2015;250:126-136. doi:10.1016/j.jneumeth.2015.01.010

© 2025 Zhukovsky P et al. *JAMA Network Open*.
