# Supplementary material for: Generalizability of Treatment Outcome Prediction Across Antidepressant Treatment Trials in Depression
Source: JAMA Netw Open. 2025 Mar 20;8(3):e251310. doi: 10.1001/jamanetworkopen.2025.1310 (PMC11926635; doi:10.1001/jamanetworkopen.2025.1310)
Supplement: Supplement 2. — Data Sharing Statement [file jamanetwopen-e251310-s002.pdf]

Data Sharing Statement

Zhukovsky. Generalizability of Treatment Outcome Prediction Across Antidepressant Treatment Trials in Depression. *JAMA Netw Open*. Published March 20, 2025. doi:10.1001/jamanetworkopen.2025.1310

Data
Data available: Yes
Data types: Deidentified participant data
How to access data: CANBIND data are available to researchers and can be accessed if a data request is approved by the Canadian Biomarker Integration Network in Depression investigators and Ontario Brain Institute. EMBARC data are publicly available at https://ndar.nih.gov,study #2199. All data are deidentified. Data available include the study protocol, data dictionary, scalar versions of clinical, behavioural, and imaging modalities, as well as raw behavioural and imaging files.
When available: With publication

Supporting Documents
Document types: None

Additional Information
Who can access the data: Researchers whose proposed use of the data has been approved
Types of analyses: Any analysis
Mechanisms of data availability: Wth a signed data access agreement
